# *In Vivo* and *In Vitro* Approaches to Modeling Hypoplastic Left Heart Syndrome

**DOI:** 10.1007/s11886-024-02122-6

**Published:** 2024-09-28

**Authors:** Matthew Alonzo, Javier Contreras, Jakob Bering, Ming-Tao Zhao

**Affiliations:** 1 Center for Cardiovascular Research, Abigail Wexner Research Institute, Nationwide Children’s Hospital, Columbus, OH 43215 USA; 2 The Heart Center, Nationwide Children’s Hospital, Columbus, OH 43215 USA; 3 Department of Pediatrics, The Ohio State University College of Medicine, Columbus, OH 43210 USA; 4 Dorothy M. Davis Heart and Lung Research Institute, The Ohio State University Wexner Medical Center, Columbus, OH 43210 USA

**Keywords:** Hypoplastic left heart syndrome, Congenital heart disease, Human induced pluripotent stem cells (iPSCs), Animal models, CRISPR/Cas9, Cardiomyocytes, Endothelial cells

## Abstract

**Purpose of Review:**

Hypoplastic left heart syndrome (HLHS) is a critical congenital heart defect characterized by the underdevelopment of left-sided heart structures, leading to significant circulatory challenges, and necessitating multiple surgeries for survival. Despite advancements in surgical interventions, long-term outcomes often involve heart failure, highlighting the need for a deeper understanding of HLHS pathogenesis. Current *in vivo* and *in vitro* models aim to recapitulate HLHS anatomy and physiology, yet they face limitations in accuracy and complexity*.*

**Recent Findings:**

*In vivo* models, including those in chick, lamb, and mouse, provide insights into hemodynamic and genetic factors influencing HLHS. *In vitro* models using human induced pluripotent stem cells offer valuable platforms for studying genetic mutations and cellular mechanisms.

**Summary:**

This review evaluates these models' utility and limitations, and proposes future directions for developing more sophisticated models to enhance our understanding and treatment of HLHS.

## Introduction

Hypoplastic left heart syndrome (HLHS) is a severe congenital heart defect characterized by the malformation of the left-sided structures of the heart, including the ascending aorta and aortic/mitral valves, resulting in an abnormally small left ventricle. This condition leads to irregular circulation, necessitating a series of open-heart surgeries to restore blood flow [1]. Despite advancements in surgical techniques that have improved the prognosis for patients with HLHS, palliation remains an imperfect solution, as many of these patients eventually develop heart failure [2]. To improve outcomes for these patients, it is crucial to understand the mechanisms underlying the onset and progression of HLHS, which will be critical to identifying novel therapeutic targets, assessing the long-term consequences of palliation, and potentially curing HLHS. Lack of robust experimental models to accurately mimic HLHS anatomy and pathophysiology, complex inheritance, phenotypic spectrum, and possible environmental contributions is impeding progress. In this review, we evaluate the current state of *in vivo* and *in vitro* models of HLHS, discuss their potential utility and limitations, and outline future directions for improving models to advance the field and ultimately improve patient outcomes.

## *In vivo* Models of HLHS

Understanding the full extent of insults that lead to the development of HLHS and the ability to test the efficacy of new surgical interventions is challenging without robust disease models that recapitulate HLHS physiology and hemodynamics. Satisfying this need will require *in vivo* models that faithfully recapitulate human cardiac physiology and hemodynamics. Although no single model of HLHS perfectly addresses both genetic and mechanical factors that play a role in the development of human HLHS, different systems provide insight into the underlying mechanisms and possible future interventions.

Early HLHS models relied on mechanical induction of left heart hypoplasia through suturing of the left atrioventricular canal to simulate impaired left ventricular inflow from mitral valve obstruction [3, 4]. Left atrial ligation (LAL) of ED4 chick hearts induce lower systolic blood pressures and myocardial stresses that fail to stimulate cardiomyocyte growth, resulting in lowered LV myocardium proliferation and volume [5, 6]. The altered hemodynamics also contribute to increased myocardium hypoxia and subendocardial collagen deposition in both ventricles [7], simulating development of endocardial fibroelastosis seen in human HLHS patients. Single-cell RNA-sequencing (scRNA-seq) of left ventricular heart tissue in ED6.5 LAL hearts showed upregulated gene expression related to fibrosis, fibroelastosis, myosin contractility, and stress-sensing in cardiomyocytes. Attempts to rescue the disease phenotype in LAL chick hearts through partial clipping of the right atrium increase left ventricular blood flow and pressure, stimulate myocardial proliferation, and increase ventricular myocardium volume [5]. Modeling a human HLHS-like phenotype in chick through altered hemodynamics provides an established platform to investigate interventions towards HLHS prevention or treatment, with study of the interactions between blood flow and myocardial growth recovery acting as a significant first step.

Subsequent lamb models of HLHS that aimed to recapitulate HLHS through implantation of a left atrial coil in mid-gestation fetal lambs to impair left ventricular inflow similarly induced left ventricular hypoplasia. Bulk RNA transcriptomics of ascending aorta tissue show altered gene expression associated with cell growth and proliferation. Single-nucleus RNA transcriptomics of left ventricular wall samples show higher fibroblast population proportion, lower cardiomyocyte proportions, and upregulation of extracellular matrix-related biological processes [8]. Although LV hypoplasia was successfully induced, significant apoptosis was not observed; further, lack of severe fibrosis indicates that endocardial fibroelastosis was not recapitulated in this model [8]. HLHS modeling in mice through mitral valve obstruction by microinjection of shear thinning biomaterial into the left atrium in E14.5 embryos showed retrograde aortic arch flow, non-apex-forming left ventricles, and ascending aortic hypoplasia [9]. While authors did not report on endocardial fibroelastosis or aortic valve anomalies, this mouse model is notable as embolized fetuses survived to term, allowing disease progression to be followed longer. Both lamb and mouse HLHS models similarly recapitulate left ventricular hypoplasia from mitral valve obstruction, supporting the chick HLHS model conclusions that limited left heart flow contributes to HLHS-like phenotype development. They also differentiate themselves from chick as large and small mammal models of left heart hypoplasia that offer testing of HLHS interventions at mid-gestation and beyond. 

While there are multiple reports using mechanical flow obstruction to induce HLHS, genetic factors are known to play a role in human HLHS development. A genetic role was hinted at by Tworetzky et al. (2004) who used aortic stenosis dilation to treat fetal aortic stenosis in 21 to 29-week gestation human fetuses predicted to develop HLHS, but found that 6 of the 14 fetuses that had undergone successful in-utero treatment still developed HLHS at term despite experiencing mitral and aortic growth [10]. However, models of genetic induction of HLHS are much scarcer. Mutagenesis screening of 3,000 mice for an HLHS phenotype identified SAP130 and PCDHA9 homozygous mutants, called the Ohia mutant line, with the highest (26%) HLHS penetrance. Further, CRISPR/Cas9 gene edited mice with double homozygous mutations in the SAP130/PCDHA9 alleles display a HLHS-like phenotype: Increased apoptosis, decreased cardiomyocyte proliferation, and altered mitochondrial maturation. The reduced LV growth in the Ohia mutant lines were attributed to SAP130 [11], as PCDHA9 homozygous mutant mice develop normal LV sizes with isolated aortic hypoplasia, stenosis, and bicuspid aortic valves. However, aortic valves anomalies may be attributed to PCDHA9. PCDHA gene mutations are enriched in human whole-exome sequencing of a cohort with 68 HLHS subjects, suggesting that PCDHA gene mutations may lead to increased BAV risk [11].

Reports of SAP130 involvement in HLHS are promising with murine SAP130 homozygous mutants displaying isolated LV hypoplasia, and knockdown of SAP130a in zebrafish causing a small ventricle phenotype [11]. However, the specificity of SAP130 mutations to left ventricle hypoplasia is unclear as mice with SAP130 homozygous mutants display isolated aortic hypoplasia with normal-sized LV and murine SAP130 heterozygous mutants show no heart defects [12]. Porcine SAP130 heterozygous mutants display primarily tricuspid dysplasia and atresia without left heart or aortic hypoplasia [13]. Interestingly, Ohia heterozygous models have lower fetal weight, which may be driven by altered placental development with reduced fetal capillary density in the placentas, lower expression of nutrient transporters, and reduced placental labyrinth region area. Placental abnormalities could contribute to the HLHS phenotype and possibly relate to embryonic lethality in Ohia SAP130; PCDHA9 homozygous mutants during gestation, thus limiting model application to human HLHS [12]. While mixed phenotypes and low disease penetrance from SAP130 knockdown suggest that genetic factors of HLHS remain unestablished, this model could serve as a starting point for developing an improved model to investigate genetic contributions to HLHS development.

Overall, these animal models provide various methods of recapitulating HLHS phenotype (Table 1). These models could allow for the testing of treatments to monitor their impact on cardiac physiology, development, and HLHS disease development. Despite the species-specific differences between models, mid-gestation impairment of left heart blood flow was repeatedly used to recapitulate an HLHS-like phenotype with models recapitulating HLHS features including left heart hypoplasia, ascending aorta hypoplasia, and increased endocardial collagen deposition. Multiple animal models of HLHS with left heart hypoplasia suggest that abnormal hemodynamics are the primary cause of HLHS, with cardiomyocyte proliferation and fibroblast collagen deposition impacted by hypoxia and myocardial stress. However, genetic models of HLHS are beginning to provide insight into the significant genetic contributors to left heart hypoplasia such as flow-independent impaired proliferation, valve development, placental development, and nutrient transportation. Complementary use of mechanical and genetic HLHS models would be a start towards addressing the inherent genetic differences of animal models. Additionally, this approach may close knowledge gaps of structural defects and cell-specific contributors of left heart hypoplasia that could lead to improved HLHS interventions. While there are limitations associated with each model, the ability to manipulate models and investigate the biological processes involved in the development of HLHS is essential to build fundamental knowledge of genetic and environmental factors that lead to HLHS phenotypes.

**Table 1:** *In vivo* models of HLHS with main findings

***In Vivo* Model**		**Main Findings**	**Reference**
Mouse	SAP130^-/-^; PCDHA9^-/-^ double homozygous mutants from CRISPR-Cas9 gene editing	• *Ohia *HLHS embryonic hearts displayed LV hypoplasia, hypoplastic aortic arch, stenotic mitral valve, and mitral valve hypoplasia at E14.5. • HLHS phenotype penetrance in SAP130^-/-^; PCDHA9^-/-^ at 26%. • Cardiomyocytes in *Ohia* HLHS LV had lower proliferation, increased apoptosis, and smaller, elongated mitochondria.	[11]
Mouse	SAP130^+/-^; PCDHA9^+/-^ double heterozygous mutants	• SAP130^+/-^; PCDHA9^+/-^ mutants had no reported heart defects, and placentas had decreased capillary density, placental labyrinth region area, and lower mRNA expression of placental angiogenesis genes PGF and KDR.	[12]
Mouse	Embolism of left atrium	• Embryos positively embolized at ED14.5 displayed non-apex-forming LV, smaller ascending aorta volume, and larger pulmonary trunk volume at ED18.5. • Positively embolized embryos that survived to gestational term displayed left heart hypoplasia and retrograde aortic arch flow at 100% penetrance.	[9]
Lamb	Left atrium coil implantation	• Fetuses with left atrial coils implanted at 0.52 gestation displayed impaired aortic valve flow, non-apex-forming LV, and left heart hypoplasia at 0.84 gestation. • Hypoplastic LVs of coiled fetuses had increased sub-endocardium thickness, collagen deposition, and upregulated expression of extracellular matrix components in fibroblasts, cardiomyocytes, and endothelial cells. • Hypoplastic LV myocardium had increased fibroblast proportion and lower cardiomyocyte proportion.	[8]
Chick	Left atrial appendage ligation	• ED4 LAL hearts displayed apex-forming RV and smaller LV at ED6, ED8, and ED12. • Increased periostin and collagen I deposition in the ED12 LV sub-endocardium, suggesting endocardial fibroelastosis (EFE) formation. • Hypoxia in the interventricular septum and compact myocardium of the ventricles at ED8 that contributes to EFE formation.	[7]
Chick	Left atrial appendage ligation	• Chicks with LAL performed at ED3.5 displayed increased LV myocardial thickness attributed to underloaded LV with lower myocardial stress and active tension. • LV myocardium of LAL hearts at ED6.5 displayed upregulation of mechano-sensing, contractility, calcium signaling, and ECM remodeling genes.	[6]
Chick	Left atrial appendage ligation + Right atrial clipping	• ED4 LAL followed by right atrial clipping at ED8 increased blood flow and preload of the LV at ED9 compared to nonclipped LAL hearts. • Right atrial clipping of LAL hearts increased cardiomyocyte proliferation in the LV compared to nonclipped LAL hearts.	[5]
Pig	SAP130+/-heterozygous mutants from CRISPR-Cas9 gene edit	• SAP130+/-live-born piglet heart defects include tricuspid valve dysplasia, tricuspid valve atresia, oval fossa atrial septal defect, and dextrocardia. • Left heart hypoplasia and aortic hypoplasia was not reported in this model.	[13]

*HLHS* hypoplastic left heart syndrome, *LV* left ventricle, *ED* embryonic day, *LAL* left atrial ligation, *RV* right ventricle, EFE endocardial fibroelastosis, *ECM* extracellular matrix

## *In vitro* Models of HLHS

*In vitro *studies aimed at understanding the pathogenesis of HLHS have primarily utilized human induced pluripotent stem cells (hiPSCs) differentiated into cardiac lineages, and more recently, on CRISPR/Cas9-mediated genetic manipulation of HLHS-associated pathogenic variants. These methodologies offer several significant advantages over traditional animal models. One advantage is that hiPSCs retain the genetic background of their donor, making them a powerful tool to study pathogenic variants within a human genetic context [11]. Additionally, hiPSCs can be differentiated into various cardiac cell populations, providing a versatile platform for detailed investigation of cardiac development and disease mechanisms. When hiPSC technology is combined with CRISPR/Cas9 gene editing, it becomes possible to engineer cardiac cells with specific mutations linked to HLHS, allowing researchers to model and study the effects of these mutations in a controlled genetic context. CRISPR/Cas9 gene editing also enables the correction of pathogenic variants in cells derived from HLHS patients, offering insight into potential therapeutic strategies. This review presents research conducted using hiPSCs as an *in vitro* laboratory model to study HLHS, highlighting the transformative potential of these technologies in unraveling the complexities of congenital heart disease.

One notable study demonstrated genetic modification of the *MYH6*-R443P pathogenic variant in both non-HLHS and HLHS-derived hiPSCs resulted in altered contractile properties of differentiated cardiomyocytes [14]. Specifically, cardiomyocytes differentiated from CRISPR/Cas9 edited healthy non-HLHS control hiPSC lines with the pathogenic *MYH6*-R443P variant had decreased sarcomeric organization with increased variant allele frequency. This gene dosage-dependent effect also led to a reduced efficiency in cardiomyocyte generation. Conversely, correcting the heterozygous *MYH6*-R443P variant in the proband using CRISPR/Cas9 rescued the dysmorphic cardiomyocyte cytoskeletal architecture. Early cardiac cells derived from HLHS hiPSCs exhibited significanlty slower shortening and relaxation rates compared to cardiomyocytes derived from the unaffected parent. This pattern was also observed in a *MYH6*-R443P variant-engineered hiPSC line, implicating the role of *MYH6 *on normal human cardiomyocyte morphology, functionality, and differentiation efficiency. Interestingly, cardiac tissue sections from HLHS samples with the *MYH6*-R443P variant displayed disrupted sarcomere structures in atrial cardiomyocytes, but not in the venticle, nor in tissue samples from HLHS patients without the *MYH6*-R443P variant or healthy donors. Taken together, this genetic and cellular model proposes a potential mechanism into the pathogenesis of *MYH6*-related HLHS via sarcomere disorganization that likely causes decreased atrial contractility and promotes hypoplastic left ventricular development. 

Among the genes implicated in HLHS, *NOTCH1* has been the most extensively studied as it has been closely associated with ventricular hypoplasia [15] and left-sided valve defects [16]. A genetic knockout of *NOTCH1* in hiPSCs using CRISPR/Cas9 technology has shown *NOTCH1* is dispensable for mesoderm formation, as cardiomyocytes and endothelial cells could still be differentiated from *NOTCH1*-null hiPSCs [17]. The deficiency of *NOTCH1* also downregulates cardiac proliferative programs that lead to stunted proliferation of hiPSC-CM *in vitro*. Despite the ability to produce cardiac and endothelial cell phenotypes from *NOTCH1*-null hiPSCs, single-cell RNA sequencing indicates shifts in cellular subtype specification during cardiomyocyte differentiation. Specifically, epicardial and second heart field progenitor cell populations were enriched in knockout cell lines at the expense of first heart field progenitors. Cardiomyocyte subtype specification was also dysrupted; *NOTCH1*-null hiPSCs preferentially differentiated into atrial-like cardiomyocytes, whereas isogenic wildtype hiPSCs primarily produced ventricular-like cardiomyocytes as evidenced by transciptional, cytoskeletal, and electrophysiological profilng. While a complete knockout of *NOTCH1* has not been observed clinically, this study shows an extreme example of roles of *NOTCH1* in cardiac cell proliferation and lineage determination by maintaining the balance of cardiac progenitor cell populations and the proper specification of cardiomyocyte subtypes, which may be dysregulated in *NOTCH1*-associated HLHS. 

Defects in NOTCH signaling were also observed in HLHS-derived hiPSC-CMs [18]. Diseased hiPSCs from patients with HLHS exhibited reduced efficiency in generating spontaneously beating hiPSC-CM cultures, and those that did differentiate displayed a lower beating rate compared to those derived from healthy donors. Transcriptional analysis of differentiating cultures revealed that HLHS hiPSCs had a diminished capacity to differentiate into the mesodermal lineage and, consequently, cardiac progenitor cells compared to healthy controls. Conversely, HLHS hiPSCs demonstrated an increased propensity to differentiate into smooth muscle cells. Ultrastructural examination of these derived cardiomyocytes showed irregular sarcomere structures with poorly defined Z bands. Exome sequencing of hiPSC-CMs identified deleterious mutations in NOTCH signaling pathways, suggesting that these key mutations may contribute to HLHS phenotypes. Functional activation of NOTCH signaling using Jagged peptide significantly enhanced cardiomyocyte generation in diseased hiPSCs while reducing smooth muscle formation. This study underscores the crucial role of NOTCH signaling in disease-specific hiPSCs in cardiac lineage generation and ultrastructure. Efficiency in HLHS-hiPSC-CM differentiation has consistently been shown to be compromised, affecting the proper formation of the cardiac mesoderm, cardiac progenitors, and subtype specification of cardiomyocytes (19). Directed differentiation of hiPSC from healthy and HLHS donors have also consistently shown disorganized sarcomere ultrastructure in HLHS-derived cardiomyocytes compared to controls. This defect in the cytoskeletal struture has reproducibly manifested as poor contraction in diseased cells and in a lower efficiency in cardiac cell generation. Corroborating results across multiple research groups seems to hint at a common *in vitro* phenotype in HLHS-hiPSC-CM.

Cardiac cells differentiated from HLHS-hiPSCs were found to have intrinsic contractility defects compared to those sourced from healthy individuals [19]. When hiPSC-CMs were cultured in micropatterned arrays, cellular contraction force and acceleration were diminished in HLHS samples, with no apparent sarcomere disorganization. scRNA-seq revealed heart-failure-related genes and pathways were enriched in HLHS hiPSC-CM compared to healthy samples, while genes related to mitochondrial and metabolic function were downregulated. HLHS cardiomyocytes also displayed reduced mitochondrial respiration and oxidative metabolism evident from reduced oxygen consumption. When treated with a high fatty acid diet, the contractility of HLHS hiPSC-CM improved. Collectively, this study demonstrates the feasability of using hiPSC-CM from HLHS patients as a platform to study heart failure, which is common in this population. It further demonstrates that cells derived from patients with HLHS display intrinsic contractility and metabolic defects compared to those derived from healthy individuals, consistent with previous reports. Another study using HLHS-hiPSC-CM demonstrated that early heart failure is associated with increased apoptosis, defects in mitochondrial respiration, and redox stress due to abnormal mitochondrial permeability transition pore (mPTP) opening and a failed antioxidant response [20]. In contrast, iPSC-CM from patients without early heart falure exhibited normal respiration and an elevated antioxidant response. Single-cell RNA transcriptomics further supported that heart failure is linked to mitochondrial dysfunction and endoplasmic reticulum stress. These findings suggest that uncompensated oxidative stress is a key factor underlying early heart failure in HLHS. Significantly, mitochondrial respiration defects, oxidative stress, and apoptosis were mitigated by treatment with sildenafil, which inhibits mPTP opening and suppresses endoplasmic reticulum stress. Collectively, these findings underscore the utility of patient-derived iPSC-CM for modeling clinical heart failure and advancing therapeutic development.

Directed differentiation of hiPSC into cardiomyocytes has been extensively studied. However, other cell types in the heart, such as arterial endothelial cells (AECs), may also contribute to the pathogenesis of HLHS [21]. Gene ontology analysis from single-cell RNA-seq of HLHS fetal cardiac tissue samples has highlighted defects in endothelial cell junction organization and migration. In arterial endothelial cells, transcriptional defects were identified in endothelium development, endothelial cell proliferation, artery morphogenesis, and NOTCH signaling pathways.

Further investigation confirmed that AECs derived from HLHS-hiPSC showed impaired proliferation *in vitro*, evidenced by reduced expression of Ki67, a marker of cell proliferation, which was also observed in diseased fetal cardiac tissue samples. These AECs also displayed similar defects in NOTCH signaling molecules as those found in HLHS fetal heart tissue. Notably, the protein level of KMT2D, a regulator of NOTCH signaling, was reduced in HLHS coronary arteries. Interestingly, treating HLHS-hiPSC-derived arterial cells with NOTCH signaling ligands increased their proliferation. This suggests that the KMT2D-mediated NOTCH defect contributes significantly to the abnormalities in coronary AECs in HLHS. The NOTCH-related defects were more pronounced in coronary AECs compared to other cardiac cell types. Disruption of NOTCH signaling led to impaired proliferation and maintenance of arterial characteristics in HLHS-hiPSC-AECs, which may partially explain the decreased vascular density and coronary artery malformations observed in the HLHS fetal heart. Notably, the application of NOTCH ligands rescued HLHS-associated gene expression abnormalities and cellular phenotypes, indicating a potential therapeutic target. 

Endocardial defects have also been shown to contribute to HLHS [22]. Single-cell RNA profiling of hiPSC-derived endocardium and human fetal heart tissue with an underdeveloped left ventricle has identified developmentally impaired endocardial populations in HLHS. Intrinsic endocardial defects contribute to abnormal endothelial-to-mesenchymal transition, disrupted NOTCH signaling, and improper extracellular matrix organization, all of which are crucial factors in valve formation. These endocardial abnormalities result in reduced cardiomyocyte proliferation and maturation by disrupting fibronectin-integrin signaling. Such intrinsic endocardial defects highlight a critical role of the endocardium in the etiology of HLHS. The association between endocardial defects and decreased cardiomyocyte proliferation and maturation points to a broader impact on heart development beyond structural anomalies.This understanding opens new avenues for potential therapeutic interventions. By targeting the endocardial function, it may be possible to develop regenerative strategies aimed at mitigating the defects seen in HLHS. Together, these results reveal the critical role of the endocardium in HLHS etiology and underscore the necessity of considering endocardial function in the development of regenerative therapies. Patient iPSC models of HLHS provide a comprehensive framework for understanding the complex molecular interactions involved in HLHS and highlights the potential for targeted therapeutic strategies to improve outcomes of HLHS.

In summary, *in vitro* models of hypoplastic left heart syndrome have primarily utilized human induced pluripotent stem cells derived from both healthy donors and patients with HLHS (Table 2). These hiPSCs have predominantly been differentiated in 2D cultures into various cardiac subtypes, particularly cardiomyocytes and endothelial cells. Additionally, some hiPSCs have been genetically engineered to either carry a HLHS-related genetic variant or have a pathogenic variant corrected. Across multiple studies, the most consistent phenotypes observed have been related to cardiac cell proliferation and differentiation, with NOTCH signaling identified as the most commonly affected and studied pathway. To advance the field, there is a need to explore the differentiation of cardiac cells using 3D models. Recent advancements in 3D cardiac organoid generation have produced more biomimetic 3D cardiac structures, providing a more accurate platform for studying cardiac diseases [23]. The ability to generate chamber-specific organoids further increases the specificity and power of these models, making them particularly valuable for investigating left-sided heart defects such as HLHS [24]. Moreover, other cardiac structures, including valve cells, fibroblasts, and other resident cell types of the heart, have been minimally studied. Given the critical role of heart valves in the pathogenesis of HLHS, greater attention should be directed towards generating and manipulating these cells from HLHS patients. Comparing their development and function to those of healthy individuals could yield significant insight. Integrating these advancements will not only refine our understanding of HLHS but also pave the way for more targeted and effective therapeutic strategies.

**Table 2:** Summary of *in vitro* models of HLHS with main findings

***In Vitro *Model**	**Main Findings**	**Reference**
CRISPR/Cas9 editing of *MYH6 *in healthy- and HLHS-derived hiPSC	• hiPSC-CM with *MYH6-*R443P variant displayed irregular sarcomere organization and contractile properties, reduced differentiation efficiency, and similar sarcomere disorganization to atrial CM from human tissue.	[14]
CRISPR/Cas9-mediated deletion of *NOTCH1* in hiPSC	• *NOTCH1* is not essential for mesoderm formation or cardiac cell differentiation. • *NOTCH1*-null hiPSC-CM display cell lineage commitment shifted cell populations to epicardial and SHF progenitors at the expense of FHF progenitors and preferentially specialized into atrial-like CM in contrast to wildtype cells that specified into ventricular-like CM and had reduced proliferation.	[17]
hiPSC from healthy donors and patients with HLHS	• HLHS hiPSC have reduced differentiation into mesoderm, cardiac progenitors, and mature cardiomyocytes but enhanced smooth muscle cells differentiation. • HLHS hiPSC-CM displayed lower beating rate, disorganized sarcomeres, and sarcoplasmic reticulum, diminished contractile force, and acceleration. • NOTCH signaling was significantly downregulated in HLHS-hiPSC-CM and activation of NOTCH by Jagged ligand restored CM generation capacity, improved beating rate, and suppressed smooth muscle cell formation. • Metabolic function was transcriptionally downregulated, and HLHS-hiPSC-CM displayed reduced mitochondrial respiration and oxidative metabolism. High fatty acid supplement improved HLHS-hiPSC-CM contractility.	[25]
Human fetal tissue and hiPSC from healthy donors and patients with HLHS	• Fetal cardiac tissue from patients showed defects in endothelial cell junction organization and migration. • Transcriptional defects in endothelium development, endothelial cell proliferation, artery morphogenesis, and NOTCH signaling pathways were observed in HLHS derived CM.	[21]
Fetal heart tissue and hiPSC from HLHS patients and healthy donors	• Intrinsic endocardial defects contribute to abnormal endothelial-to-mesenchymal transition, disrupted NOTCH signaling, and improper extracellular matrix organization, all of which are crucial factors in valve formation. • Endocardial abnormalities result in reduced cardiomyocyte proliferation and maturation by disrupting fibronectin-integrin signaling.	[22]

*HLHS *hypoplastic left heart syndrome*, hiPSC* human induced pluripotent stem cell, *hiPSC-CM* human iPSC-derived cardiomyocyte, *CM* cardiomyocyte, *FHF* first heart field, *SHF* second heart field

## Conclusion and Future Directions

**Fig. 1:**
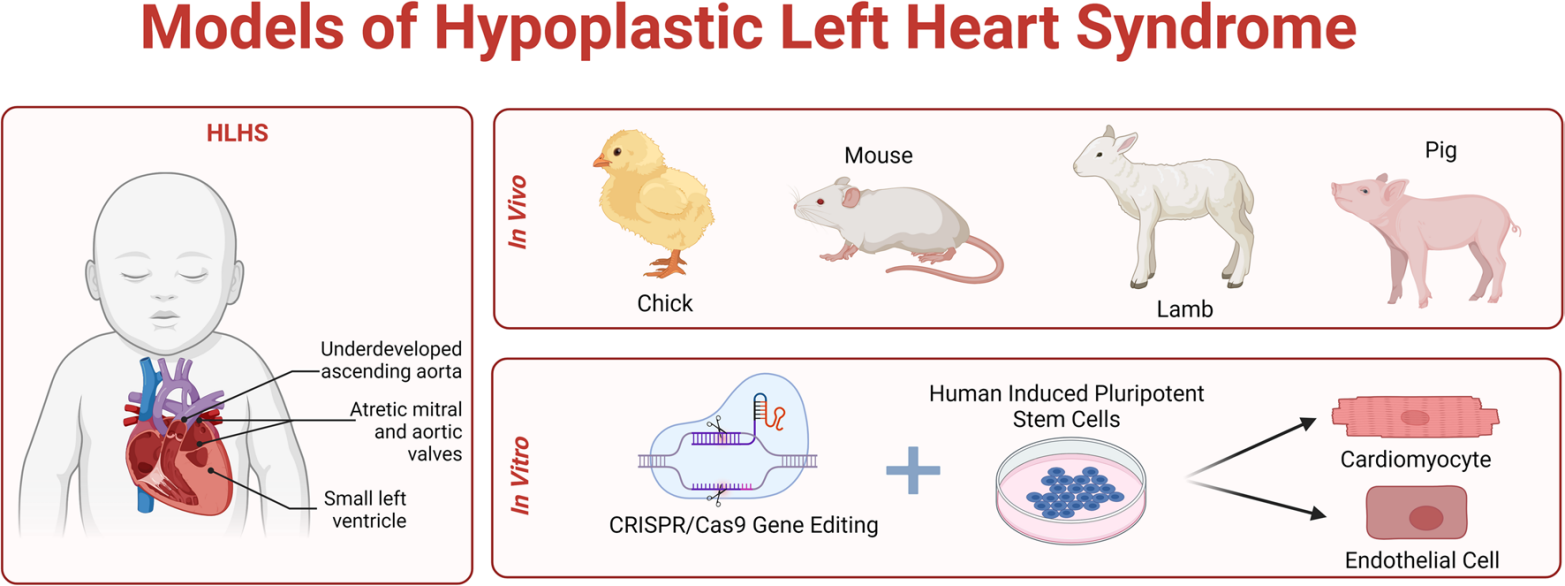
Current *in vivo* and *in vitro* models of HLHS. *In vivo* animal models include genetic or mechanical perturbations in chick, mouse, lamb, and pig. *In vitro* models include genetic manipulation of HLHS-associated variants or intrinsic defects in hiPSC-derived cardiac cells from HLHS patients

In conclusion, current *in vitro* and *in vivo* models of hypoplastic left heart syndrome have significantly advanced our understanding of this complex congenital heart defect (Fig. 1). *In vitro* models, particularly those utilizing human induced pluripotent stem cells, have provided valuable insight into the genetic and cellular mechanisms underlying HLHS. These models allow for precise genetic manipulation and differentiation into various cardiac cell types, enabling detailed studies of disease-related phenotypes and the identification of potential therapeutic targets. However, these models primarily rely on 2D cultures and have yet to fully replicate the complex 3D architecture and dynamic environment of the human heart. The recent development of 3D cardiac organoids offers a promising avenue to overcome these limitations, providing more biomimetic platforms for disease modeling and drug testing.

*In vivo* models have been instrumental in elucidating the physiological and hemodynamic aspects of HLHS. Animal models using mice, lambs, and chicks have replicated key features of disease including impaired cardiac growth and abnormal valve development. These models have been crucial for testing surgical interventions and understanding the impact of mechanical and genetic factors on HLHS progression. However, species-specific differences and incomplete recapitulation of human HLHS limit the translational potential of these models. Moreover, genetic models are still in the early stages, with only a few genetic mutations being studied.

To further enhance the utility of HLHS models, future research should focus on improving the physiological relevance and complexity of both *in vitro* and *in vivo* systems. Cell-cell interaction and cell-environment interactions are among the least studied in HLHS using current models. *In vitro* studies should prioritize the development of advanced 3D models that incorporate multiple cardiac cell types, including valve cells, fibroblasts, and endothelial cells, to better mimic the heart's cellular diversity. Additionally, integrating mechanical stimuli into these models could provide more accurate representations of cardiac function. *In vivo* research should aim to develop genetically engineered models that more accurately reflect the human condition, potentially using gene-editing technologies like CRISPR/Cas9. Combining these advanced models with comprehensive genomic and proteomic analyses will facilitate a deeper understanding of HLHS pathogenesis and aid in the identification of novel therapeutic targets.

Overall, while current models of HLHS have provided valuable insight, there is a clear need for more sophisticated and integrated approaches to fully understand and address this devastating condition. By advancing our modeling techniques and expanding our focus to include a wider array of cardiac cell types and genetic factors, we can pave the way for more effective treatments and ultimately improve outcomes for patients with HLHS.

## Key References

- Reuter MS, Sokolowski DJ, Javier Diaz-Mejia J, Keunen J, de Vrijer B, Chan C, Wang L, Ryan G, Chiasson DA, Ketela T, Scherer SW, Wilson MD, Jaeggi E, Chaturvedi RR. Decreased left heart flow in fetal lambs causes left heart hypoplasia and pro-fibrotic tissue remodeling. Commun Biol. 2023;6(1):770. 10.1038/s42003-023-05132-2. Epub 2023/07/23. PubMed PMID: 37481629; PMCID: PMC10363152.
  - Findings from this study suggest early gestation fetal mitral valve stenosis and its associated low left ventricular inflow causes left heart hypoplasia in fetal lambs similar to human HLHS.
- Ye S, Wang C, Xu Z, Lin H, Wan X, Yu Y, Adhicary S, Zhang JZ, Zhou Y, Liu C, Alonzo M, Bi J, Ramirez-Navarro A, Deschenes I, Ma Q, Garg V, Wu JC, Zhao MT. Impaired Human Cardiac Cell Development due to NOTCH1 Deficiency. Circ Res. 2023;132(2):187-204. 10.1161/CIRCRESAHA.122.321398. Epub 20221230. PubMed PMID: 36583388; PMCID: PMC9852089.
  - Findings from this study demonstrate that NOTCH1 disruption alters cardiomyocyte differentiation by inhibiting FHF progenitor generation, and leads to cardiomyocyte determination towards an atrial fate at the cost of ventricular subtype.
- Xu X, Jin K, Bais AS, Zhu W, Yagi H, Feinstein TN, Nguyen PK, Criscione JD, Liu X, Beutner G, Karunakaran KB, Rao KS, He H, Adams P, Kuo CK, Kostka D, Pryhuber GS, Shiva S, Ganapathiraju MK, Porter GA, Jr., Lin JI, Aronow B, Lo CW. Uncompensated mitochondrial oxidative stress underlies heart failure in an iPSC-derived model of congenital heart disease. Cell Stem Cell. 2022;29(5):840-55. 10.1016/j.stem.2022.03.003. Epub 20220407. PubMed PMID: 35395180; PMCID: PMC9302582.
  - Findings from this study suggest that cardiomyocytes derived from HLHS patients with heart failure have altered functionality with increased apotosis, mitochondrial respiration defects, and oxidative stress.

## Data Availability

No datasets were generated or analysed during the current study.
